# PROTAC-Based Strategies in Neurodegenerative Diseases: Challenges and Perspectives

**DOI:** 10.3390/ph19091352

**Published:** 2026-08-26

**Authors:** Pasquale Degennaro, Imane Ghafir El Idrissi, Rosa Purgatorio, Annalisa Fanizzi, Mariagrazia Rullo, Leonardo Pisani, Eleonora Macchia, Luisa Torsi, Angela Stefanachi, Francesco Leonetti

**Affiliations:** 1 Department of Pharmacy and Pharmaceutical Science, University of Bari Aldo Moro, 70125 Bari, Italy; p.degennaro8@phd.uniba.it (P.D.); imane.ghafir@uniba.it (I.G.E.I.); rosa.purgatorio@uniba.it (R.P.); annalisa.fanizzi@uniba.it (A.F.); mariagrazia.rullo@uniba.it (M.R.); leonardo.pisani@uniba.it (L.P.); eleonora.macchia@uniba.it (E.M.); 2 Department of Chemistry, University of Bari Aldo Moro, 70125 Bari, Italy; luisa.torsi@uniba.it

**Keywords:** targeted protein degradation (TPD), Proteolysis-Targeting Chimeras (PROTACs), neurodegenerative diseases, multi-target strategies

## Abstract

Proteolysis-Targeting Chimeras (PROTACs) are heterobifunctional molecules that induce the selective degradation of a protein of interest by recruiting an E3 ubiquitin ligase, thereby triggering ubiquitination and proteasomal clearance. As part of the broader targeted protein degradation (TPD) paradigm, PROTACs offer a powerful strategy to eliminate pathogenic proteins that are difficult to modulate with traditional occupancy-based inhibitors. However, their clinical translation is often limited by poor aqueous solubility, suboptimal cellular permeability, and off-target effects. Notably, some PROTACs retain potent biological activity despite limited membrane permeability, owing to their catalytic mechanism of action, which allows even a small number of molecules reaching the target site to drive substantial protein degradation and produce important pharmacological effects. Growing evidence supports the application of PROTAC-based approaches in neurodegenerative diseases, where the selective removal of toxic or misfolded proteins is particularly attractive. This review summarizes recent advances in chemical protein degradation strategies for neurodegenerative disorders and highlights potential future perspectives of multifunctional PROTACs for therapeutic development.

## 1. Introduction

The World Health Organization predicts that neurodegenerative diseases (NDs) are destined to become the second most common cause of death within the next 20 years [1]. NDs encompass a broad spectrum of clinically and pathologically diverse disorders affecting millions of individuals worldwide; regardless of the various symptoms and characteristic pathological conditions, they underlie highly similar molecular mechanisms. Indeed, the aggregation of specific proteins represents a common histopathological hallmark: misfolded proteins accumulate intracellularly and/or extracellularly into ordered structures, leading to functional and structural alterations of neurons and other cell types [2,3]. Consequently, a progressive degeneration of neurons is observed in both the central nervous system (CNS) and the peripheral nervous system [4,5].

Amyloid-β, alpha-synuclein (α-Syn), and Tau are the most representative neuropathogenic proteins, whose balance and interconnection play a crucial role in a range of neurodegenerative diseases, such as Alzheimer’s disease (AD), Parkinson’s disease (PD), and polyglutamine (polyQ) diseases [6,7,8].

In this context, the increasingly profound understanding of the underlying pathogenesis and molecular mechanisms, the emergence of novel technologies and strategies related to drug discovery within the scientific research landscape, and the limited benefits of small-molecule-based therapies have undoubtedly marked a decisive turning point in the design of new candidates for the treatment of such pathologies.

The use of small molecules for the modulation of protein targets represents the classical approach to drug discovery. Small molecules are low-molecular-weight compounds that bind to a well-defined binding site of proteins of interest (POIs), such as enzymes, ion channels, and receptors, thereby modulating their function. However, several proteins lack binding sites and catalytic activity, rendering their modulation challenging. It is estimated that over 80% of proteins are “undruggable” [9], a percentage that includes critical targets such as transcription factors (TFs), structural proteins, and non-enzymatic proteins. For these reasons, alternative therapeutic approaches have been developed over the years to address these challenges [10]. Monoclonal antibodies (mAbs) [11], antisense oligonucleotides (ASOs) [12], and small interfering RNAs (siRNAs) [13] constitute only a minor example in this regard, although several issues still limit their clinical applicability today.

**Figure 1:**
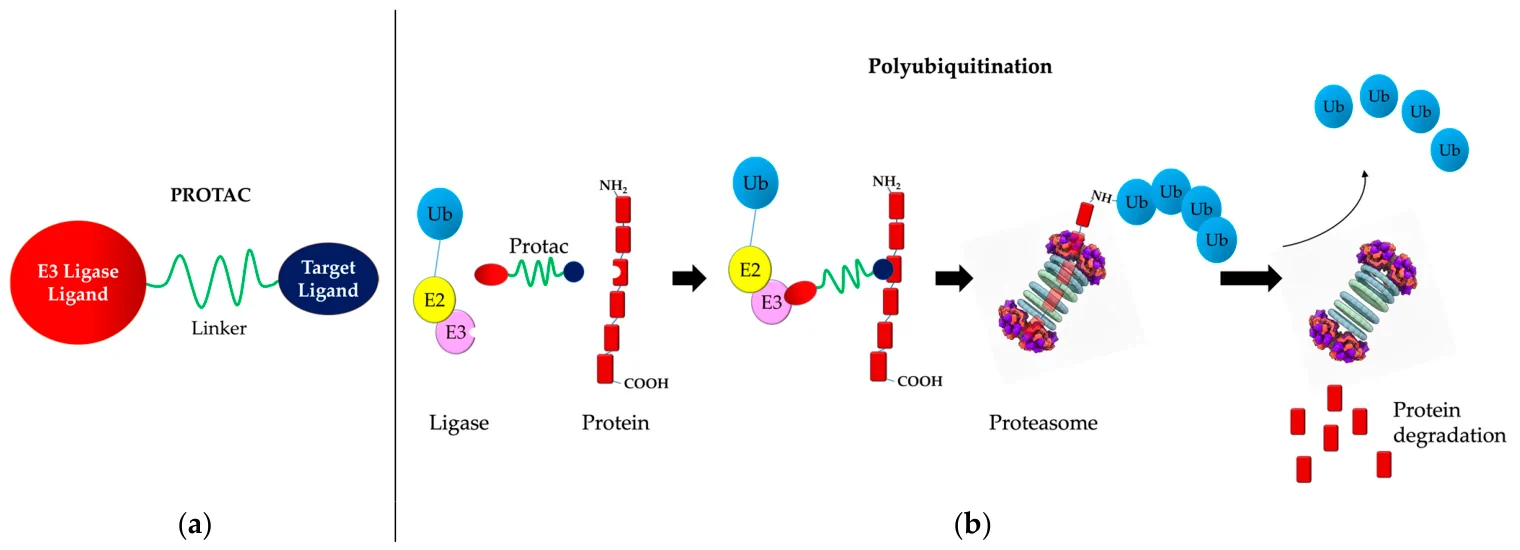
(**a**) Generic representation of PROTAC structure, (**b**) PROTAC mechanism of action.

Despite the common perception that small molecules have now run their course, they continue to form the backbone of pharmaceutical research. Undoubtedly, small molecules have fostered the development of a revolutionary way of conceptualizing drugs, based on PROTACs. Through this innovative approach, small molecules—acting as building blocks of PROTACs—are utilized to control protein levels [14,15]. Indeed, PROTACs do not inhibit the protein of interest, but they induce its degradation by exploiting the cellular ubiquitin–proteasome system (UPS) (Figure 1b). As such, they intervene in the complex process employed by cells to regulate the concentration, conformation, and localization of proteins: protein homeostasis [16,17].

PROTAC-mediated protein degradation therefore represents an extraordinary strategy to enhance classical drug discovery approaches, offering the opportunity to target “undruggable” proteins [18]. However, their poor cell permeability severely limits their applicability. As such, the incorporation of cell-penetrating peptide (CPP) sequences was the first approach used for improving the delivery of PROTAC molecules in living cells [19].

It must be mentioned that while PROTACs are highly modular and customizable for a broad spectrum of targets and endowed with suboptimal drug-like properties, at present, molecular glues are gaining prominence in targeting the ubiquitin–proteasome system.

Molecular glues are monovalent, low-molecular-weight small molecules, typically under 500 Daltons, that lack dual-binding heads or synthetic linkers. They act allosterically by reshaping the substrate-binding interface of an E3 ligase to induce or stabilize new protein–protein interactions. A key example is lenalidomide, which binds cereblon to create an interaction surface that recruits and degrades the transcription factors IKZF1 and IKZF3. Due to their compact size, lower polar surface area, and fewer hydrogen bond donors and acceptors, molecular glues usually offer superior drug-like properties, high membrane permeability, strong oral bioavailability, and an enhanced ability to cross the blood–brain barrier. However, because most molecular glues are discovered through phenotypic screening, their rational, structure-based design remains challenging [20]. 

To expand beyond the ubiquitin–proteasome system, which primarily degrades soluble intracellular targets, researchers are tapping into the lysosomal pathway. The lysosome offers a major degradation route capable of clearing a much broader range of targets, including membrane proteins, extracellular entities, and large protein aggregates, thereby bypassing the limits of traditional inhibitors and proteasomal degraders.

Several emerging technologies leverage this lysosomal route. Lysosome-targeting chimeras, or LYTACs, are bifunctional molecules that cross-link target proteins or cell-surface receptors with cell-surface lysosome-targeting receptors, such as the cation-independent mannose 6-phosphate receptor, driving the internalization and degradation of extracellular and membrane-bound pathogenic proteins. Autophagy-targeting chimeras, or AUTACs, feature specific chemical tags like S-guanylation mimics that trigger selective autophagy; the target is engulfed into an autophagosome that fuses with a lysosome, making AUTACs effective at clearing intracellular protein aggregates, larger cellular structures, and dysfunctional organelles like mitochondria. Similarly, autophagosome tethering compounds, or ATTECs, are small molecules that directly bind both the target protein and LC3 on the autophagosomal membrane, efficiently capturing non-soluble toxic aggregates and routing them directly to the lysosome [21].

## 2. A Brief History of PROTAC Development

**Figure 2:**
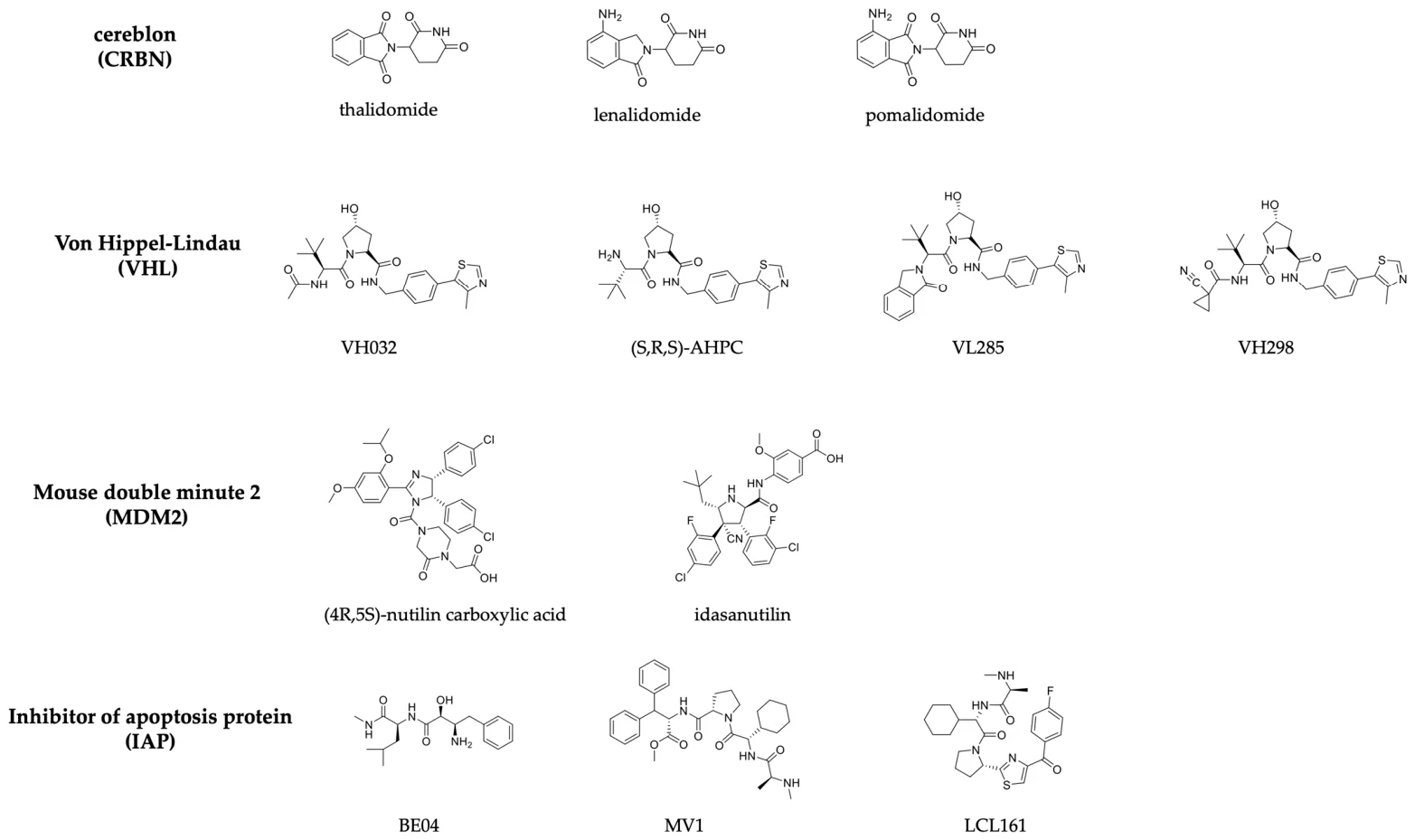
Chemical structure of representative E3 ligase ligands in PROTACs.

The typical structure of a PROTAC molecule is composed of a tandem of functional components: (1) a ligand target protein of interest (POI); (2) a ligand engaging an E3 ligase (Figure 2); and (3) a linker that connects the two elements (Figure 1a). 

The first PROTAC was reported by Crews and colleagues in 2001, utilizing a phosphopeptide chain to recruit the β-TrCP (β-Trasducin Conteninig Protein) E3 ligase [22]. In 2008, the first all-small-molecule PROTAC based on the Mouse double minute 2 (MDM2) as the E3 ligase (Figure 2) and selective androgen receptor modulators (SARMs) was reported [23]. In 2010, the use of thalidomide derivatives as molecular glues based on the Cereblon moiety (CRBN) was identified [24]. Between 2012 and 2014, several PROTACs based on the von Hippel–Lindau moiety (VHL) and CRBN were synthesized (Figure 2) [25,26].

The physicochemical profile of PROTACs places them beyond the Rule-of-Five (bRo5) space, usually unfavorable for efficient blood–brain barrier (BBB) penetration. Small molecules, owing to their greater potential for optimization of drug-like properties and oral bioavailability, represented a decisive turning point in the development of this technology, attracting increasing interest from both the scientific community and the pharmaceutical industry [27].

Indeed, since 2017, an exponential growth in scientific publications related to PROTACs has been observed.

In 2019, Arvinas Therapeutics initiated the first Phase I clinical trials for ARV-110 [28] for the treatment of metastatic prostate cancer and ARV-471 [29] for the treatment of advanced or metastatic breast cancer—two orally bioavailable degraders targeting the androgen receptor (AR) and the estrogen receptor (ER), respectively. Thanks to the excellent results obtained in Phase I, the two compounds entered Phase II in 2020 and 2022, respectively [30,31]. In 2023, ARV-471 continued its progression in the VERITAC-2 Phase III clinical trial [32,33]. To date, over 20 types of PROTACs for the treatment of various cancers have entered clinical trials.

In 2024, Arvinas initiated a Phase I clinical trial for ARV-102, a novel PROTAC designed to selectively degrade the protein leucine-rich repeat kinase 2 (LRRK2) [34]. This PROTAC is an orally available compound capable of crossing the blood–brain barrier and is the only PROTAC that has progressed to clinical phase studies for the treatment of neurodegenerative diseases.

**Figure 3:**
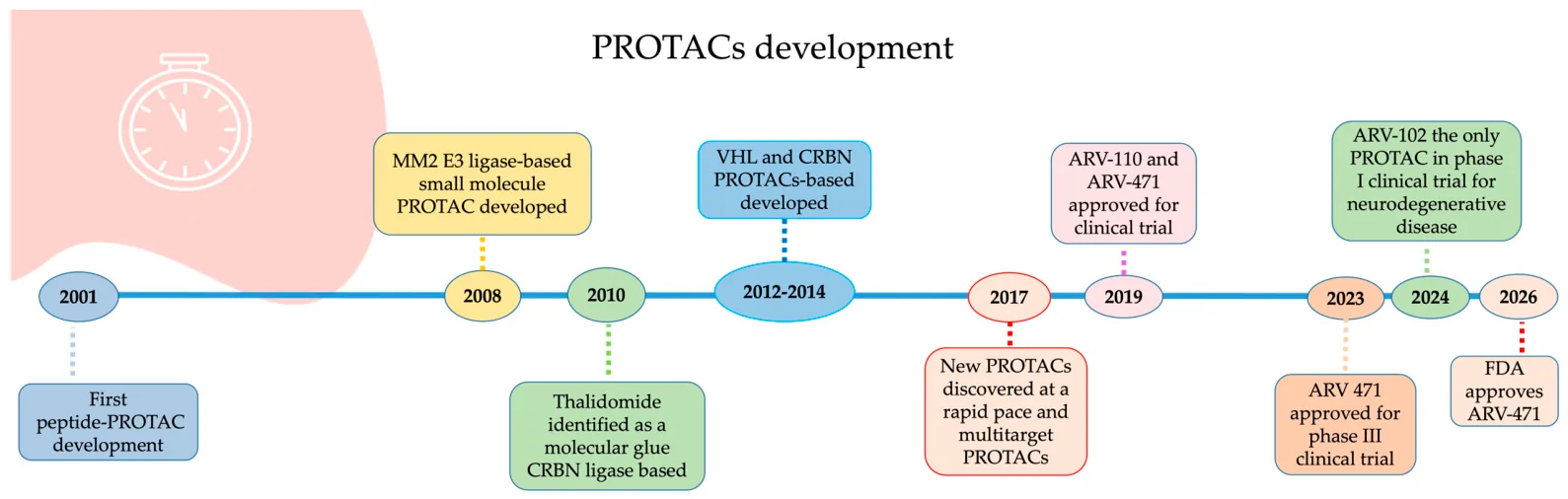
PROTAC timeline from the first PROTAC peptide to the first one that was FDA-approved.

In May 2026, ARV-471 received FDA approval for the treatment of adults with estrogen receptor-positive (ER+), HER2-negative, ESR1-mutated advanced or metastatic breast cancer [35]. This approval constitutes a landmark event and represents, for the entire scientific community and for drug discovery, the culmination of a journey that began in 2001 and meticulously progressed until the anticipated results were achieved (Figure 3).

Over the years, increasingly detailed knowledge has been acquired regarding alternative approaches to classical drug design, and the PROTAC toolbox has expanded to the point of now including PROTACs endowed with a second mode of action. The incorporation into the classical PROTAC architecture of molecular fragments capable of conferring additional functionality, alongside proteasome-mediated degradation, has led to the development of “multifunctional PROTACs” [36]. Depending on the newly introduced functionality, these systems may involve polypharmacology, photopharmacology, drug conjugates, macrocycles, or oligonucleotides. This review will focus specifically on the polypharmacology approach. 

**Figure 4:**
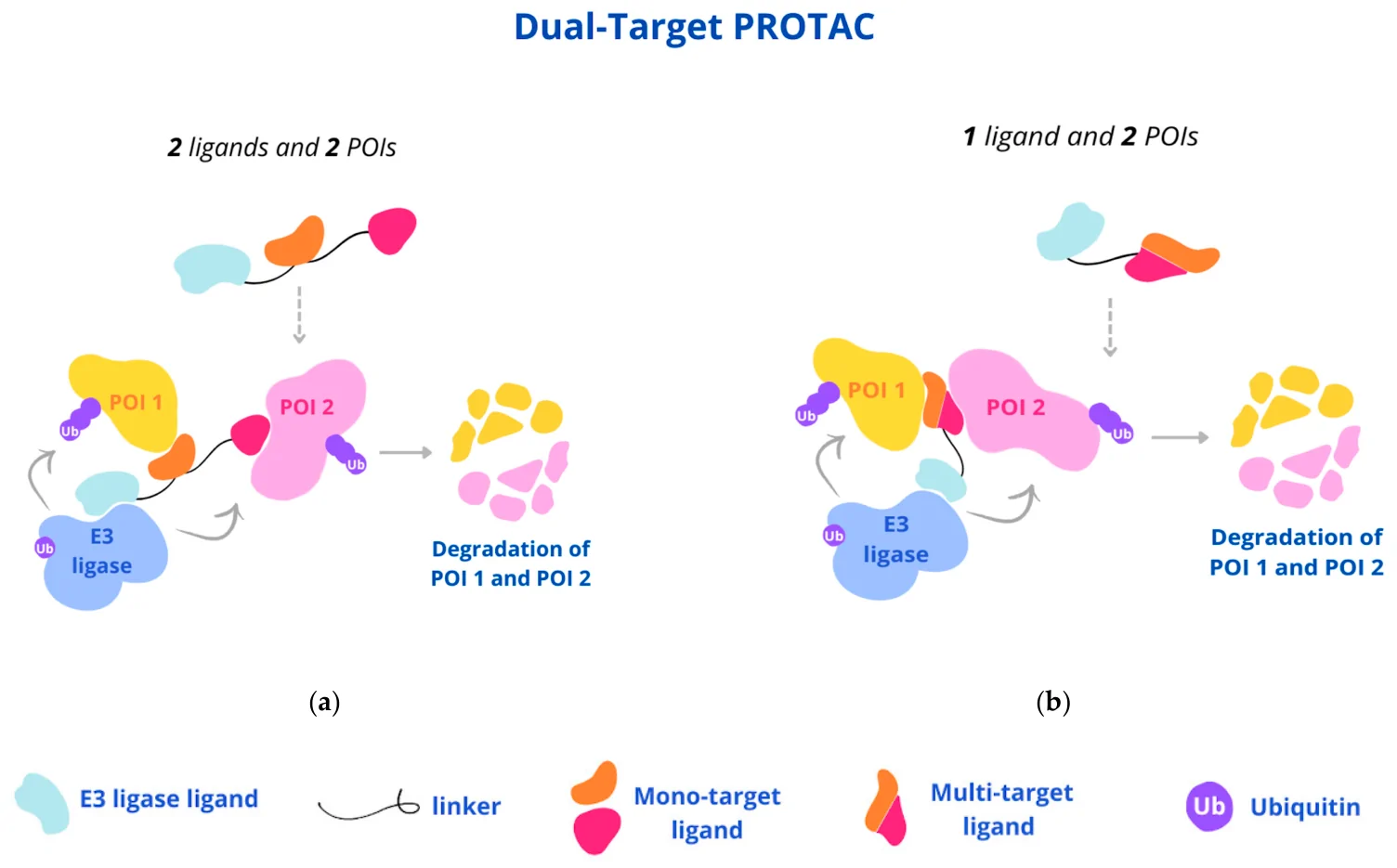
Mechanism of action of multi-target PROTACs: (**a**) two different protein of interest (POI) ligands binding to two different POIs and (**b**) one dual-targeting POI ligand binding to two different POIs.

Complex diseases, such as cancer and neurodegenerative disorders, are regarded as the result of dysregulation within physiological networks; therefore, it is unlikely that a single intervention—for example, using conventional single-target drugs—can fully restore the altered condition. By contrast, the simultaneous modulation of multiple targets may contribute to achieving a successful therapeutic outcome. Polypharmacology encompasses the use of pharmaceutical agents known as MTDLs (multi-target-directed ligands), which are multi-target drugs that have become a cornerstone of modern medicinal chemistry. MTDLs are single molecules with multiple mechanisms of action, developed through the combination of parent scaffolds [37]. The implementation of this approach within PROTAC technology is particularly advantageous owing to the large number of experimental and/or approved drugs displaying multi-target profiles. Representative examples include serotonin–dopamine activity modulators, such as Aripiprazole, approved for the treatment of psychiatric disorders; and dual cyclin-dependent kinase inhibitors, such as Palbociclib, used in the treatment of complex cancers. Within the PROTAC field, the multi-target approach, intended as the concerted pharmacological modulation of two or more targets, may be achieved through two different strategies: the PROTAC incorporates two distinct mono-target POI ligands (Figure 4a), or the PROTAC incorporates a single POI ligand endowed with dual or multiple activity (Figure 4b) [36].

Recently, a library of PROTACs endowed with two distinct mono-target POI ligands was reported by Zheng and co-workers [38]. In this study, gefitinib and olaparib were combined into CRBN- or VHL-based PROTACs with the aim of degrading two interconnected targets involved in cancer progression, namely the epidermal growth factor receptor (EGFR) and poly(ADP-ribose) polymerase (PARP). These PROTACs contain a linker connecting two independent POI ligands and an E3 ligase ligand. Among them, the CRBN-based PROTAC DP-C-1 displayed the most favorable dual degradation profile, outperforming the corresponding mono-PROTACs at the same concentration. 

Although multi-target PROTACs have demonstrated improved modulation of pathological networks compared with mono-target compounds, multi-target PROTACs based on two distinct POI ligands represent a greater challenge than conventional PROTACs from both a synthetic and a pharmacokinetic perspective, owing to their increased structural complexity. 

Regarding chemical synthesis, dedicated strategies and “click chemistry platforms” have been developed. In particular, the “click chemistry” reaction involved a copper-catalyzed azide–alkyne cycloaddition between an azide-functionalized target ligand and an E3 ligase ligand bearing a terminal alkyne, yielding a novel triazole linker. This strategy provides an efficient approach for parallel synthesis and facilitates the discovery and optimization of new PROTACs, thus confirming the continued interest in this class of compounds [39,40].

From a pharmacokinetic (PK) perspective, the presence of a second POI ligand increases the molecular weight relative to traditional PROTACs, thereby posing additional challenges in terms of cellular permeability and/or oral bioavailability. In the case of PROTACs containing a dual POI ligand, the multi-target activity is generally directed toward proteins belonging to the same family, as they share a certain degree of similarity in their binding sites. Conversely, integrating within a single structure of molecular fragments displaying affinity for highly distinct binding sites is considerably more difficult. In the field of neurodegenerative diseases, considering the limited and non-curative therapeutic efficacy of currently available drugs, PROTACs undoubtedly represent an attractive and highly promising strategy, since these diseases are frequently associated with aggregates of misfolded proteins considered to be undruggable targets. A brief description of the principal targets investigated in PROTAC studies, as well as those for dual PROTACs, is provided below.

## 3. PROTACs in Neurodegenerative Disease

### 3.1. PROTACs in Alzheimer’s Diseases

The Tau protein is a microtubule-stabilizing protein that is essential for maintaining neuronal structure and function. In Alzheimer’s disease (AD), Tau undergoes abnormal hyperphosphorylation, leading to the formation of neurofibrillary tangles that impair axonal transport and ultimately cause neuronal dysfunction and death [41].

**Figure 5:**
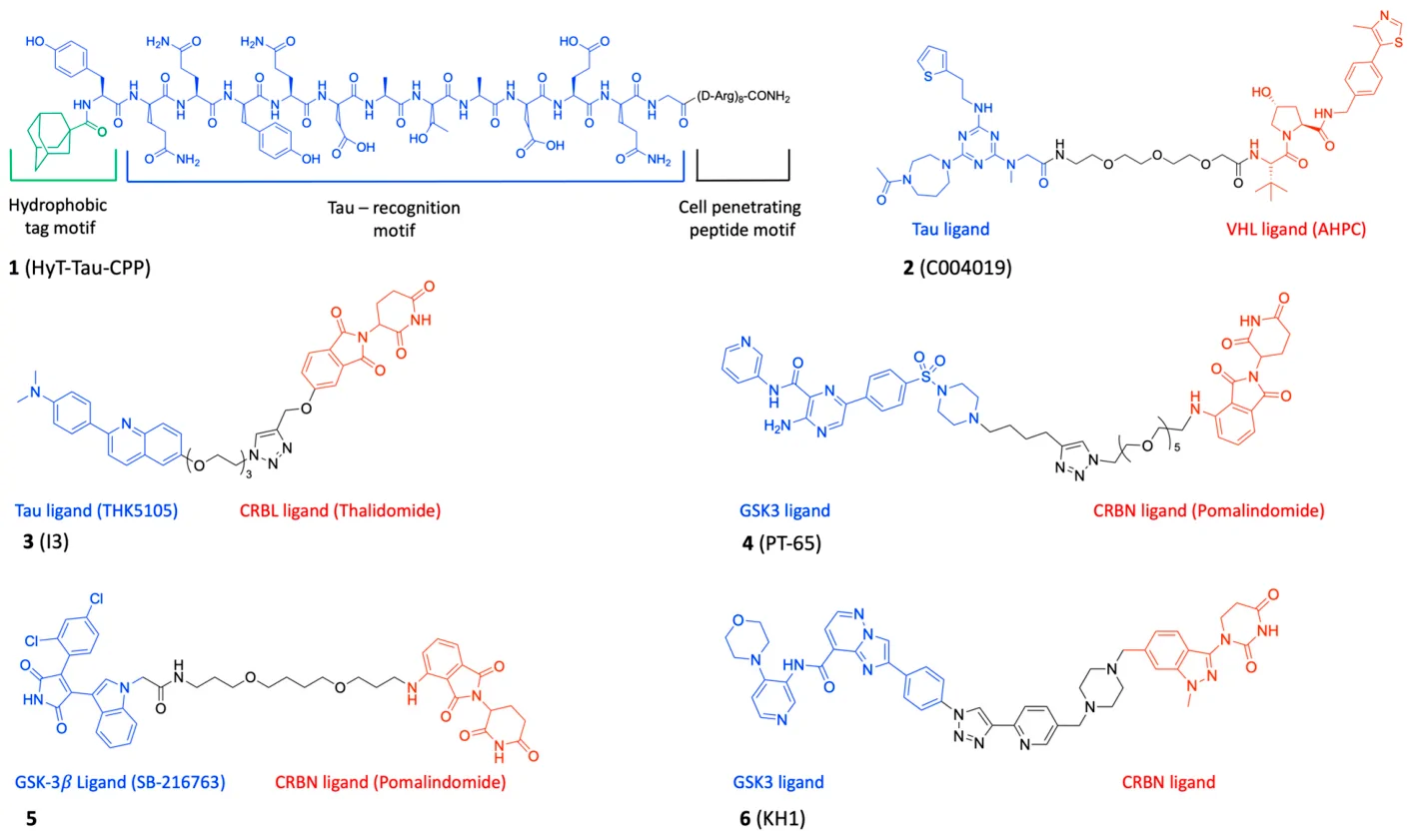
Structure of compounds active against AD targets. Hydrophobic tag motif (green), POI ligand (blue), linker (black), E3 ligase ligand (red).

In 2017, the Li group reported a series of hydrophobic tagging (HyT)-based degraders targeting Tau proteins [42]. Hydrophobic tagging (HyT) represents an alternative degradation strategy that exploits the cellular protein quality control system, wherein heat-shock proteins (HSPs) recognize exposed hydrophobic residues on misfolded proteins and target them for degradation. This concept was generalized by the Crews group through the development of HyT, which coupled a hydrophobic adamantyl moiety to a ligand for the protein of interest, thereby inducing HSP-mediated proteolysis [43]. Following this approach, the Li group developed compound **1** (named HyT-Tau-CPP in the original publication; see Figure 5), which is constituted by three motifs: the “Tau-recognition peptide motif” as warhead, the adamantyl group as the “hydrophobic tag motif”, and the “cell-penetrating peptide motif” (CPP, constituted by eight arginine residues). Compound **1** effectively reduced intracellular Tau levels in a proteasome-dependent manner. In fact, tau-EGFP-overexpressing cells were incubated with different concentrations (0–150 μM) of compound **1**; the Tau level was evaluated by flow cytometry assay or Western blot and subsequently quantified in ESI, and the maximal degradation level was 80% in a dose-dependent manner (Table 2). Notably, intravenous administration of this HyT-Tau-CPP compound (**1**) resulted in significant Tau degradation in the brains of AD model mice (3xTg-AD); in particular, the Western blot analysis showed a reduction in Tau in the hippocampus and cortex regions. Moreover, these findings demonstrate that compound **1** is able to efficiently cross the cell membrane. This observation was further corroborated by flow cytometry analysis in N2a cells, which revealed a significant increase in intracellular fluorescence intensity following treatment with a carboxyfluorescein-labeled analog of compound **1** [42]. 

In 2021, Wang and colleagues designed and synthesized compound **2** (named C004019 in the original publication; see Figure 5), a PROTAC molecule directed against Tau protein with VHL as the E3 ligase, enhancing Tau protein ubiquitination and proteolysis. Compound **2** was able to induce a significant increase in Tau clearance in the HEK293 and SH-SY5Y cell lines. In particular, compound **2** is able to reduce Tau protein with an IC_50_ of 0.00785 μM in HEK293-hTau cells (Table 2), and to confirm the ubiquitination mode of action, the simultaneous treatment of HEK293 cells with MG132 (a proteasome inhibitor) was performed. The co-treatment with MG132 abolished the clearance of Tau, confirming the ubiquitination and proteolysis of Tau by the proteasome pathway. Moreover, after intracerebroventricular infusion, the hippocampus level of Tau decreased in the brains of 3xTg-AD and wild-type mice. To identify whether compound **2** could penetrate the blood–brain barrier, the concentration of compound **2** in plasma and in the brain was calculated. The data showed a maximal concentration of 10.8 ng/ mL at 0.167 h with t _1/2_ of 1.29 at 3 mg/kg of subcutaneous administration and, at the maximal concentration, a brain/plasma ratio of 0.00866. These data showed that compound **2** could induce robust clearance of Tau even though the brain concentration was low [44].

In 2022, Huang and colleagues developed a series of compounds designed by conjugating the PET tracer THK5105, a 2-arylquinoline derivative with affinity for Tau, to a thalidomide-based E3 ligase ligand via a PEG linker. Among them, compound **3** (named I3 in the original publication; see Figure 5), bearing a PEG linker of three units, showed activity as a degrader of Tau protein in a dose-dependent manner and was also able to reduce Aβ-induced cytotoxicity in PC12 cell lines. Pharmacokinetic studies of the compound revealed a brain-to-plasma concentration ratio exceeding 1.6 in healthy rats at 30 mg/kg (po) administration, indicating favorable brain penetration [45].

Amyloid-beta (Aβ) is a peptide derived from the amyloid precursor protein through cleavage mediated by β-secretase and γ-secretase. In AD, Aβ peptides aggregate to form extracellular amyloid plaques, disrupting intercellular communication while simultaneously triggering immune responses and inflammation, ultimately resulting in neuronal damage. However, the pathological alterations caused by Tau accumulation could be considered to occur downstream of Aβ aggregation within the pathogenic cascade of the disease. Consequently, targeting Tau protein may represent a possible therapeutic strategy in neurodegenerative diseases such as Alzheimer’s disease [41].

Glycogen synthase kinase 3 (GSK-3) is an enzyme that catalyzes the phosphorylation of proteins on serine/threonine residues. In particular, the GSK-3β isoform, predominantly localized in the central nervous system, is responsible for Tau phosphorylation. Numerous studies have shown that elevated GSK-3β levels are associated with a substantial reduction in the generation of new neurons within the hippocampus of patients affected by Alzheimer’s disease compared with healthy individuals [46,47]. This explains the use of GSK-3β inhibitors as ligands in PROTAC design; indeed, the reduction could restore the level of neurons in the hippocampus of AD patients. In 2021, using a click chemistry platform, Wang developed a series of GSK-3 PROTACs, among which compound **4** (named PT-65 in the original publication) showed the best activity as a GSK-3 degrader in the nanomolar range (DC_50_ = 28.3 nM for GSK3α and DC_50_ = 34.2 nM for GSK3β, Table 2) in SH-SY5Y cell lines. Moreover, compound **4** reduced the hyperphosphorylation of Tau in cell and in vivo models of AD (Figure 5). Compound **4** was analyzed for pharmacokinetic properties; the experimental data (PAMPA, Pe = 0.27 × 10^−6^ cm/s) and calculated values (LogP_o/w_ = 1.61 and Bioavailability score = 0.17; Swiss ADME) showed a moderate-to-low brain penetration [48].

In 2023, the Milelli group designed and synthesized new GSK-3 degraders, using two different GSK-3 inhibitors: SB-215763 and tideglusib linked to pomalidomide as the E3 ligase [49]. Among them, compound **5** (with SB-215763 as a warhead) emerged as the most effective GSK-3β degrader with a DC_50_ of 6.22 μM (Table 2), non-toxicity at 20 μM in neuronal cells, and the ability to reduce the neurotoxicity induced by Aβ_25-35_ in the SH-SY5Y cell line. Notably, compound **5** also exhibited moderate BBB permeability (effective permeability: Pe = 15.33 ± 1.12 × 10^−6^ cm/s, PAMPA-BBB assay), supporting its potential utility in CNS-oriented applications. 

In 2025, Farnaby and colleagues [50] investigated and applied a concept of using orthogonally reactive linker reagents, which allowed them to construct screening libraries varying the E3 ligase ligand, the target protein ligand and the linker simultaneously, and moreover, to test compounds directly in cells. PROTAC potency, kinetics, and absorption, distribution, metabolism and excretion (ADME) profiles are all influenced by the choice of ligands, linkers and conjugation chemistry used. To cover a broad physicochemical property space, aromatic, heteroaromatic, rigid moiety, PEG-based, and aliphatic compounds were used as linkers; the classic CRBN, VHL and MDM2 were used as E3 ligands; and GSK3-targeting binders were selected as the POIs. Among them, compound **6** (named KH1 in the original paper; see Figure 5), bearing the imidazo[1,2-b]pyridazine as the GSK-3β-recruiting moiety, showed DC_50_ values in the picomolar to single-digit nanomolar range for degrading both GSK-3 isoforms (Table 2). Moreover, after in vivo mice administration (5 mg kg^−1^, i.v.) and within 4 h, compound **6** was capable of complete elimination of GSK-3β in the liver and partial degradation thereof in the brain, highlighting the rapid degradation kinetics profile and moderate brain penetration. In fact, the pharmacokinetic study performed with compound **6** on Balb/c mice displayed low but measurable plasma and brain concentrations (oral bioavailability of 1.6%, plasma concentration above 88 nM, brain concentration of 16 nM and a brain/plasma ratio of 0.18, at 2 h following i.v. dosing at 0.37 mg/kg). Collectively, these findings underscore the robustness of orthogonally reactive linker chemistry and support a direct-to-biology approach as an effective strategy to accelerate the discovery of CNS-active PROTACs.

### 3.2. PROTACs in Parkinson’s Disease 

**Figure 6:**
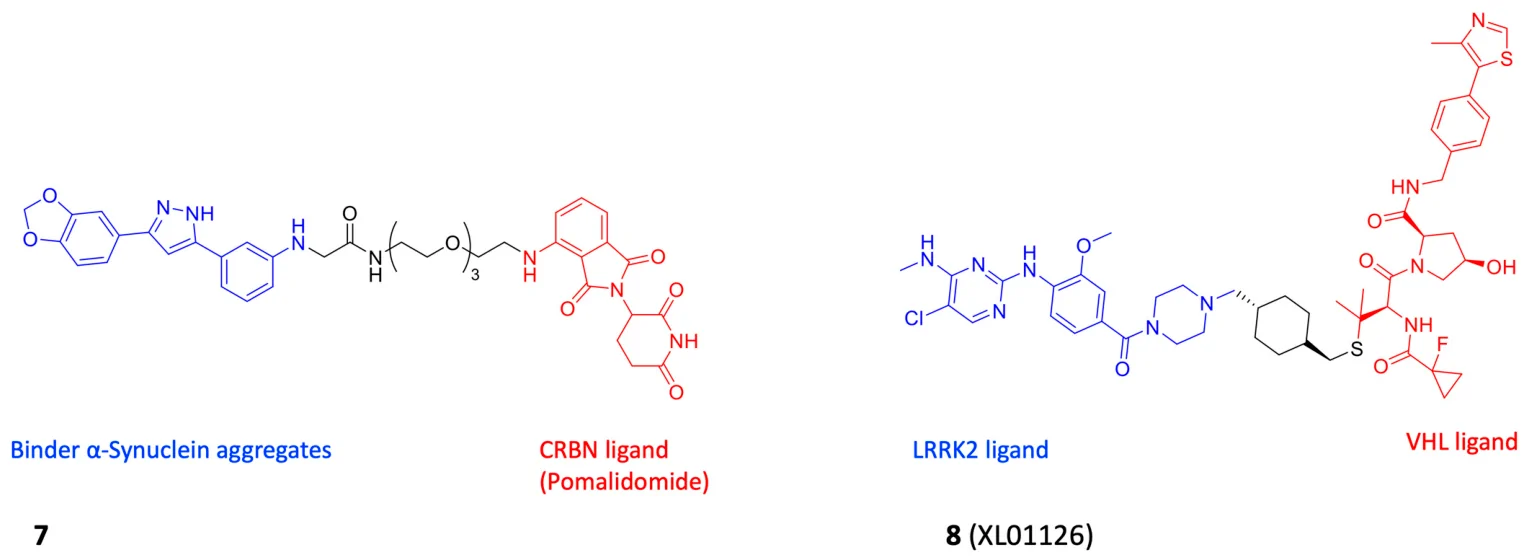
Structure of compounds active against PD targets. POI ligand (blue), linker (black), E3 ligase ligand (red).

α-Synuclein (α-syn), a protein composed of 140 amino acids, is widely recognized as a critical factor in the pathogenesis of Parkinson’s disease (PD), owing to its tendency to misfold and form intracellular aggregates known as Lewy bodies [51,52,53]. The abnormal accumulation of α-syn within neurons leads to neurodegeneration; therefore, emerging therapeutic strategies are focused on reducing α-syn levels. In 2023, Pang and colleagues reported a series of small molecules capable of degrading α-syn aggregates, and among them, compound **7** (bearing a binder of α-syn aggregates as a warhead) [54] showed the highest activity with a DC_50_ value of 7.51 μM and a D_max_ value of 89% in HEK293T cells (Table 2, Figure 6). Moreover, the lipophilicity of compound **7** was measured by logP via HPLC (log P = 2.044), and the cell penetration was monitored by harnessing its auto-fluorescence effect by confocal LSM with 10 μM of compound **7** on HEK293T cells (live-cell imaging). The results indicated that compound **7** has good membrane-penetrating ability.

Leucine-rich repeat kinase 2 (LRRK2) is a large multidomain protein possessing both kinase and GTPase activity, and it plays a key role in the pathogenesis of PD. Mutations in the LRRK2 gene, particularly the G2019S mutation, result in increased kinase activity, thereby activating neuronal cell death signaling pathways [55,56].

In 2022, Ciulli and colleagues reported the study and synthesis of compound **8** (named XL01126 in the original publication), a PROTAC degrader of LRRK2 based on VHL as the E3 ligase (Figure 6) [57]. This compound exhibited rapid, potent, and selective degradation activity, with DC_50_ values ranging from 15 to 72 nM across multiple cell lines, D_max_ values between 82% and 90%, and degradation half-lives of 0.6–2.4 h (Table 2). Compound **8** represented a significant advancement among PROTAC molecules because it demonstrated high cellular permeability. Following a single administration of compound **8** via intravenous (IV, 5 mg/kg), intraperitoneal (IP, 30 mg/kg), or oral gavage (PO, 30 mg/kg), its concentrations were determined in plasma, brain tissue, and cerebrospinal fluid (CSF). High plasma concentrations were achieved regardless of the route of administration, and compound **8** was also detected in both brain tissue and CSF at levels exceeding its DC_50_. These findings indicate that compound **8** is orally bioavailable and capable of crossing the blood–brain barrier [57].

The only compounds that have progressed to Phase I clinical studies are ARV-102, a leucine-rich repeat kinase 2 (LRRK2, IC_50_ = 0.14 nM) [58] degrader developed for the treatment of Parkinson’s disease (PD) and progressive supranuclear palsy (PSP) (Table 2) [34], and ARV-027, a degrader of the polyglutamine-expanded androgen receptor (polyQ-AR) intended for the treatment of spinal and bulbar muscular atrophy (SBMA) [59].

### 3.3. PROTACs in Huntington’s Disease

Mutant huntingtin (mHtt) is the principal pathogenic factor responsible for Huntington’s disease (HD). It is an aggregation-prone protein characterized by an expanded polyglutamine (polyQ) sequence caused by an autosomal-dominant mutation in the corresponding gene. In this context, ligands must display high specificity towards the mutant form of huntingtin without affecting wild-type huntingtin, which is essential for normal cellular function [60].

**Figure 7:**
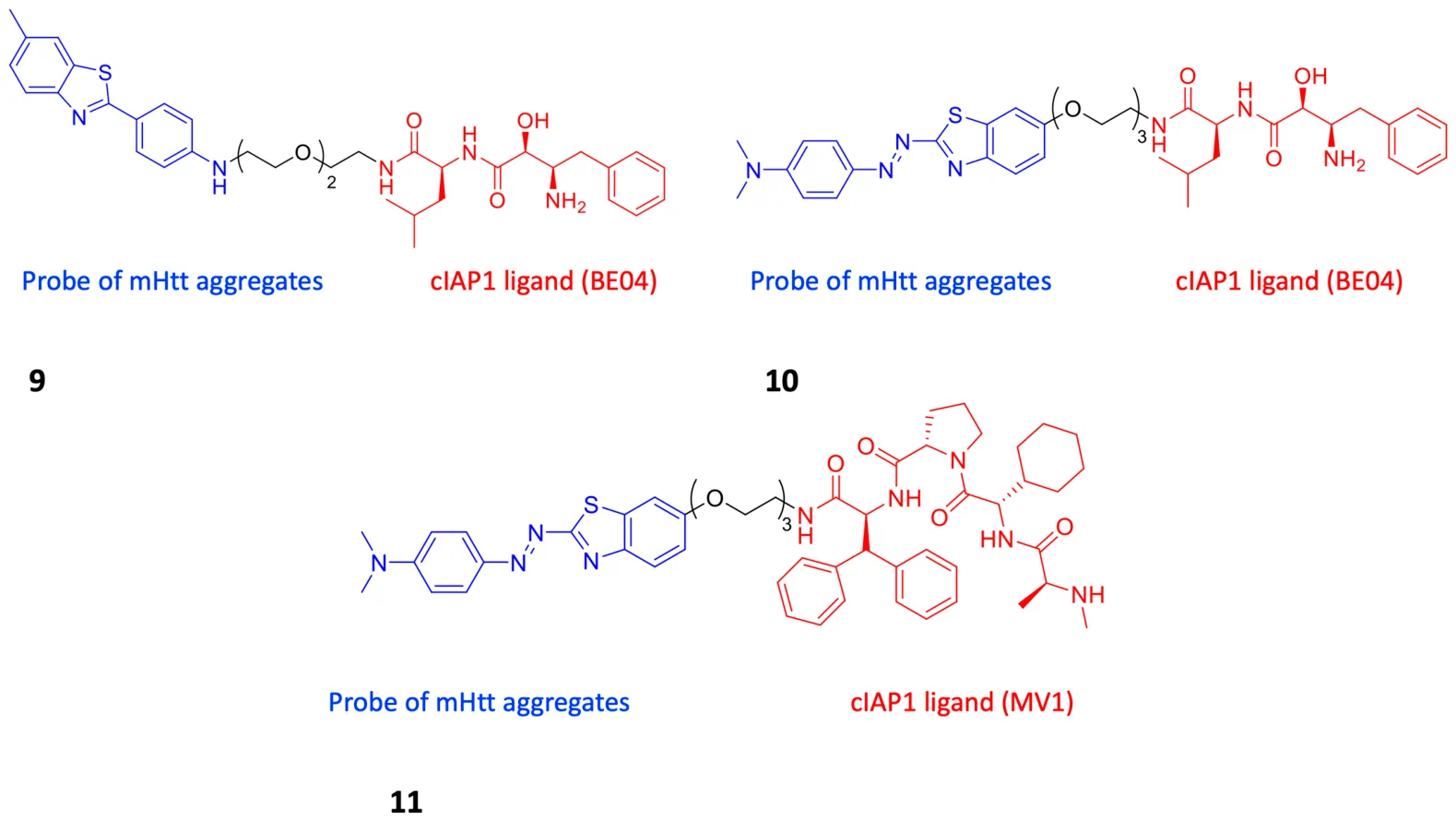
Structure of compounds active against HD targets. POI ligand (blue), linker (black), E3 ligase ligand (red).

As the analog of Thioflavin-T (ThT), 2-[4-(methylamino)phenyl]-6-methylbenzothiazole (BTA) and phenyldiazenyl benzothiazole (PDB) were used as mHtt probes for targeting the protein aggregate. In 2017, the Ishikawa group developed two important PROTACs, compounds **9** and **10** (Figure 7), bearing a BTA and PDB moiety, respectively, as the molecule that detects Htt aggregates, and combining a ligand for the ubiquitin ligase cellular inhibitor of apoptosis protein 1 (cIAP1). Compounds **9** and **10** showed efficacy, and mechanistic analysis indicated that the compounds promote the formation of a complex between Htt aggregates and cIAP1, which in turn starts the proteasomal degradation of Htt aggregates in fibroblasts derived from HD patients and in healthy subjects (Figure 7) [61].

In 2018, the same group reported the synthesis of a new Htt protein degrader, compound **11**, in which MV1 was the E3 ligase ligand in place of Bestatin-amido-methyl (BE04), distinguishing it from compound **10** (Figure 7). Notably, while MV1 showed higher affinity for IAP compared with BE04, the efficacy of compound **11** in the degradation of Htt aggregates was reduced, suggesting the importance of the linker in determining overall activity [62].

### 3.4. PROTACs in Amyotrophic Lateral Sclerosis

The nuclear TAR DNA-binding Protein 43 (TDP-43) appears to play a crucial role in the pathogenesis of Amyotrophic Lateral Sclerosis (ALS). In ALS patients, TDP-43 protein translocates from the nucleus to the cytoplasm, where it tends to aggregate and form cytoplasmic inclusions. The presence of these inclusions is believed to represent a key determinant of ALS pathology [63]. TDP-43 ligands must therefore exhibit high specificity towards misfolded or aggregated forms of TDP-43, since normal TDP-43 is essential for cellular processes.

**Figure 8:**
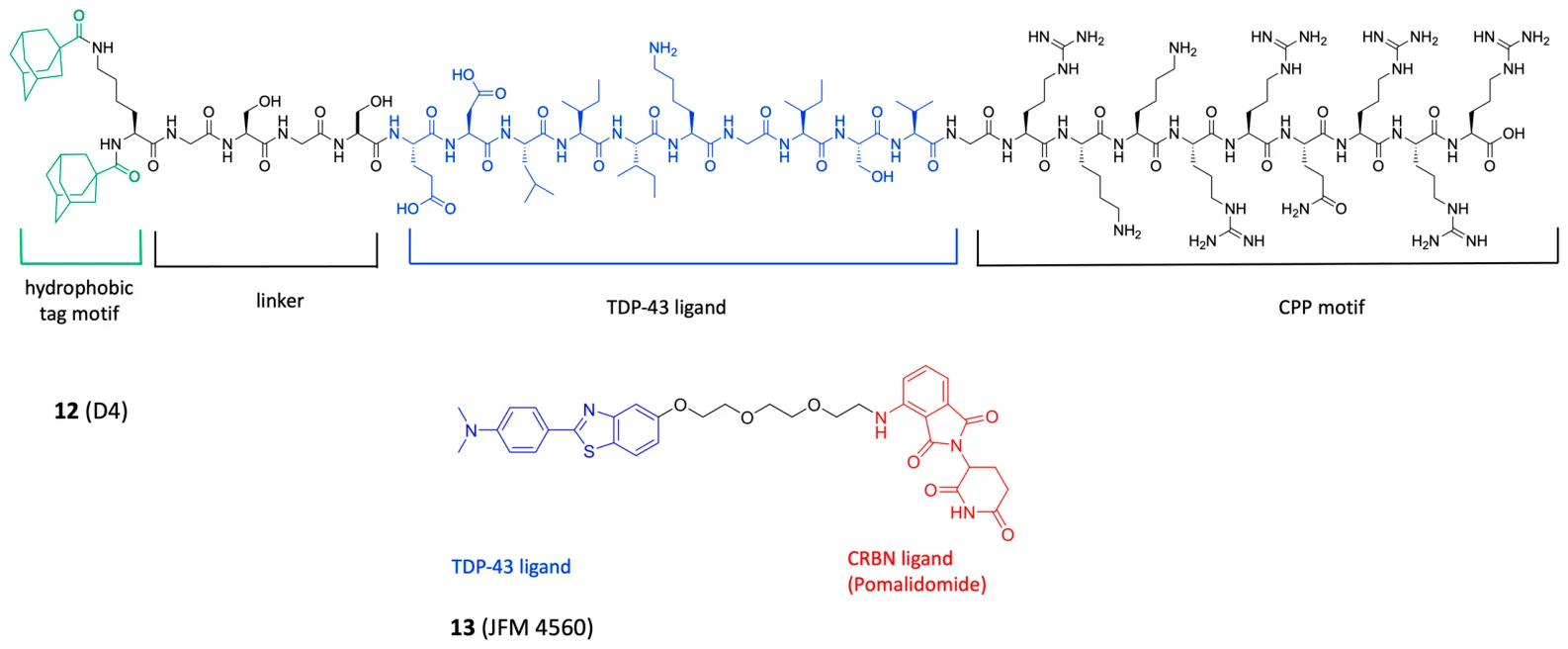
Structure of compounds active against ALS targets. Compound **12**: hydrophobic tag (green), linker (black, peptide sequence KGSGS), POI ligand TDP-43 (blue, peptide sequence EDLIIKGISV), cell-penetrating peptide motif (CPP, peptide sequence GRKKRRQRRR); compound **13**: POI ligand TDP-43 (blue), linker (black), E3 ligase ligand (red).

In 2019, the Li group designed and synthesized different single and double hydrophobic tags. Among them, compound **12** (D4, original name) displayed the highest ability to degrade TDP-43 in cells overexpressing TDP-43 (N2a), which was determined via Western blot experiments (Figure 8) [64]. Moreover, to characterize whether compound **12** was able to get into cell, a derivate of compound **12** with a fluorescent tag (carboxyfluorescein, CF) was synthesized to yield CF-12. The N2a cells were treated with 100 μM of CF-12. Flow cytometric results showed an increase in intracellular fluorescence during the time of incubation, indicating that compound **12** could penetrate into cells. In vivo studies in a Drosophila model overexpressing TDP-43 demonstrated that compound **12** also exhibits degrader activity in vivo.

In 2023, the Fang team developed four PROTACs with different linker lengths. Among these molecules, compound **13** (JMF 4560, original name) showed significant reduction in TDP-43 aggregation. To quantify the remaining levels of C-TDP-43 aggregates following PROTAC treatment, the authors performed a filter trap assay coupled with immunoblotting by loading Neuro-2a cell lysates onto a cellulose acetate (CA) membrane. The results revealed a high level of aggregates in the control sample (1.08 ± 0.31) compared with the blank (0.38 ± 0.08). Among the tested compounds, compound **13** showed the greatest reduction in aggregate levels (0.41 ± 0.06), whereas the other compounds exhibited more modest effects, with values ranging from approximately 0.64 to 1.31 (Table 2). Moreover, compound **13** enhanced cell viability in Neuro-2a cell lines against TDP-43-induced cytotoxicity (Figure 8) [65]. 

### 3.5. Dual-PROTACs in Neurodegeneration Disease: A New Prospective Approach

Analysis of the scientific literature published to date in the field of neurodegenerative diseases reveals a marked tendency towards a classical PROTAC strategy. Indeed, many of the reported libraries consist of compounds directed against a single target, presumably with the aim of ensuring highly selective activity while minimizing off-target effects. Moreover, it has long been recognized that the etiology and progression of many neurodegenerative disorders are multifactorial in nature. Consequently, it would be appropriate to expand research efforts in the field of polypharmacology, with particular emphasis on multi-target PROTACs. A similar approach has already been successfully applied in the treatment of resistant and/or advanced cancers, leading to improved outcomes compared with conventional mono-target degraders. Herein, we report the only published example of this approach in neurodegenerative diseases. 

**Figure 9:**
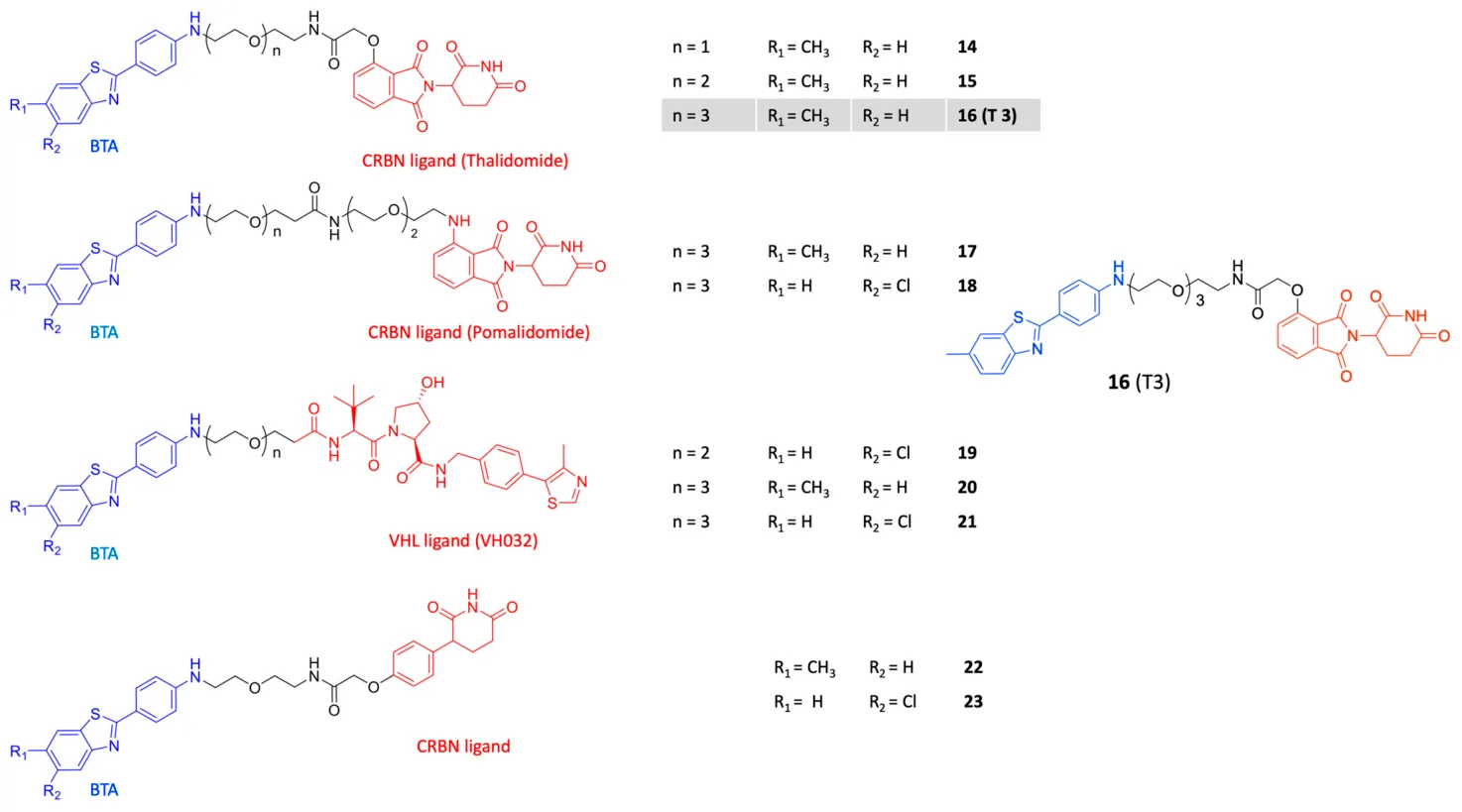
Structure of dual PROTAC. α-Synuclein and Tau fibrils ligand (BTA) (Blue), linker (black), E3 ligase ligand (red).

In 2024, the Pang group studied and synthesized a series of dual PROTAC degraders using 2-[4-(methylamino)phenyl]-6-methylbenzothiazole (BTA) as a warhead. These compounds were able to simultaneously degrade α-Synuclein (α-Syn) and Tau protein aggregates. BTA, an analog of ThT, has good brain penetration and was employed as a dual POI (protein of interest) ligand, and its binding affinity towards preformed α-synuclein and Tau fibrils was evaluated (K_D_ values of 0.32 and 2.55 μM, respectively). The linker consisted of flexible poly(ethylene glycol) (PEG)-based chains with variable length (8, 11, 14, 21 atoms), while thalidomide (compounds **14**–**16**), pomalidomide (compounds **17** and **18**), VH032 (compounds **19**–**21**) and phenylglutarimide (compounds **22** and **23**) moieties were used as E3 ligands (Figure 9). Among the series of dual PROTACs, compound **16** (original name T3; see Figure 9) demonstrated the highest activity in the degradation of α-Synuclein (α-Syn) and Tau protein aggregates and was shown to bind two proteins with dissociation constants (K_D_) of 0.47 and 2.78 μM, respectively, values very close to those of BTA [66]. Compound **16** was able to reduce α-Syn aggregates and total Tau levels in a dose-dependent manner, exhibiting DC_50_ values of 1.57 and 4.09 μM and D_max_ values of 78% and 61%, respectively (Table 2). Confocal laser scanning microscopy (LSM) images of live SH-SY5Y cells under different experimental conditions confirmed the in vitro permeability of T3. In addition, the ability of compound **16** to cross the blood–brain barrier was evaluated in vivo by fluorescence imaging of mouse brain tissue one hour after intravenous administration at different doses. Brain samples were subsequently analyzed by high-performance liquid chromatography (HPLC) and liquid chromatography–mass spectrometry (LC–MS), revealing a positive correlation between the concentration of compound **16** in brain tissue and the administered dose. Following the evaluation of blood–brain barrier (BBB) permeability through both in vitro and in vivo studies, an MPTP-induced Parkinson’s disease mouse model was used to assess the clearance of α-Syn and Tau induced by degrader **16** after intravenous administration at doses of 2, 4, and 8 mg/kg. The results indicated that administration of compound **16** at 8 mg/kg effectively reduced aggregated protein levels and protected dopaminergic neurons. Moreover, compound **16** prevented cell death due to accumulation of α-Syn by inhibiting α-Syn-aggregate-mediated ROS generation, which results in the loss of mitochondrial membrane potential. In summary, compound **16** protected neuronal cells from α-Syn and Tau aggregate-associated toxicity and was able to penetrate through the BBB. In conclusion, these dual PROTACs represent a promising therapeutic strategy for neurodegenerative diseases.

It is worth noting that, for the design of dual PROTACs targeting polymer-forming fibrils, it is necessary to assess whether monomer/polymer degradation occurs and if the ubiquitin–proteasome system can effectively achieve it. To this end, the study by Kumar et al. provides valuable insight by detailing the aggregation and fibril formation mechanisms of Aβ and Tau, as well as identifying the key amino acid residues required for effective binding of the designed PROTAC and subsequent ubiquitin-mediated degradation [8]. Alzheimer’s disease (AD) could be challenged by PROTAC-based polypharmacology strategies. Indeed, a hallmark of AD is represented by neuritic plaques due to β-amyloid (Aβ) deposits and neurofibrillary tangles made by intraneuronal aggregates of hyperphosphorylated Tau protein. Researchers in the field followed the cholinergic hypothesis, grounded in acetylcholine (ACh) depletion, suggesting acetylcholinesterase (AChE) inhibitors as a possible therapeutic strategy. As a possible alternative to this unsuccessful strategy, butyrylcholinesterase (BChE) has more recently been regarded as a viable target to develop anti-AD therapeutics. BChE can be found within glial cells, where it exerts a probable compensatory role for diminished AChE. In fact, BChE expression increases in parallel with disease progress and the enzyme co-localizes with neuritic plaques [67]. Because of the multifactorial nature of AD, researchers envisaged the so-called multi-target approach, looking for drug candidates able to control multiple targets at the same time (multi-target-directed ligands, MTDLs) that gain effective therapeutic action from their multiple activity [68]. Monoamine oxidases A and B (MAO A and B) represent challenging targets for designing MTDLs. Both isoforms are responsible for the degradation of arylalkylamines at the mitochondrial level. Since MAO-catalyzed oxidative deamination produces hydrogen peroxide (a radical initiator) and aldehyde catabolites (electrophilic species), their activity can be seen as a source of ROS. The preferential blockade of the B isoform, prevalent within the CNS, can also reduce the risk of toxicity from peripheral MAO A inhibition. Moreover, MAO inhibition can alleviate the neuroinflammation process in AD brains by limiting the production of pro-inflammatory cytokines released by reactive astrocytes overexpressing MAO B. Our idea is that a dual ChEs-MAO B PROTAC could be useful for regulating the expression of these neurodegenerative disease-associated proteins and gaining not only the inhibition but the complete degradation of target enzymes crucial for AD onset and progression, namely BChE and MAO B.

## 4. PROTACs and Pharmacokinetic Studies: Challenges Beyond the Rule of Five

As is already known, in neurological diseases, PROTACs may offer an alternative therapeutic strategy by inducing the degradation of target proteins involved in neurodegenerative disorders (NDDs). 

Nevertheless, their high molecular weight, the high number of HB donor and acceptor groups and their lipophilicity values place them in the “beyond Rule-of-Five” chemical space, which is typically unfavorable for crossing the blood–brain barrier [69].

The blood–brain barrier itself is maintained by brain endothelial cells within the neurovascular unit, supported by pericytes, astrocytes, microglia, neurons, and the basement membrane. Brain endothelial cells feature specialized tight and adherens junctions that create high transendothelial electrical resistance, severely restricting paracellular diffusion. Transport across this barrier occurs through carrier-mediated transport, receptor-mediated transcytosis, adsorptive-mediated transcytosis, or suppression mechanisms like Mfsd2a, while active efflux transporters such as P-glycoprotein, BCRP, and MRPs actively pump compounds back into the bloodstream.

To ensure adequate brain exposure, optimization leverage is built into every phase of discovery: in silico models (Pfizer’s CNS Multi-Parameter Optimization score, CNS-MPO) [70] drive initial design and in vitro assays (cell-free PAMPA-BBB, traditional 2D cell cultures, 3D organoids, emerging microfluidic organ-on-a-chip devices and others) [71] guide lead refinement, but only in vivo studies deliver definitive exposure data for advanced candidates.

Caron et al., analyzing confirmed brain-penetrant PROTACs, revealed unique physicochemical patterns. Their molecular weights range from approximately 780 to over 1000 Daltons, far exceeding traditional brain-penetration limits. To maintain permeability despite these high weights, hydrogen bond donor counts are kept strictly low, rarely exceeding four or five. In contrast, hydrogen bond acceptor numbers range from 14 to 21, resulting in topological polar surface area values that far exceed conventional guidelines [72].

Moreover, some PROTACs with very low permeability have nevertheless demonstrated significant biological activity, possibly due to their catalytic mechanism of action, which requires only a limited number of PROTAC molecules to reach the site of action in order to exert their pharmacological effect.

The literature search yielded 13 studies on neurodegenerative diseases published between 2017 and 2025, describing a total of 14 PROTAC molecules. All identified compounds, together with their in vitro and in vivo molecular targets and pharmacokinetic studies, are summarized in Table 1. 

Regarding the in vitro data for BBB permeability, three degraders (**4**, **5** and **16**) were tested using an in vitro BBB model. In particular, compounds **4** and **5** were evaluated using the PAMPA model. Moreover, compound **16** was tested in a transwell-based co-culture of brain endothelial cells (BECs) and SH-SY5Y cells, and its fluorescence emission was detected using Confocal Laser Scanning Microscopy (CLSM). Similarly, the fluorescence emission of compound **7** was assessed in HEK293T cell lines (CLSM). The cell membrane permeability assay, Caco-2, was used for analyzing compound **8**, whereas degraders **1** and **12** were tested using the flow cytometry method on neuroblastoma N2a cell lines. Compounds **5**, **7** and **12** were considered CNS-permeant following the in vitro results. PROTACs **8** and **16** were evaluated for their in vivo brain concentrations; PROTACs **2**, **3**, and **6** were tested only for in vivo permeability. The data for all of them confirm values denoting good-to-moderate BBB permeability.

No in vitro and in vivo assays have been reported for the other PROTACs (**9**–**11**, **13**).

Notably, Wang and colleagues identified the membrane receptor expressed on brain endothelial cells as a mediator for different degraders facilitating their endocytosis [73]. Moreover, nanotechnology-enabled delivery systems—including liposomes, polymeric nanoparticles, DNA nanostructures, and inorganic nanocarriers—provide promising solutions by improving pharmacokinetics, stability, and target engagement. In particular, a recent study by Xie and colleagues reported the molecular recognition and applicability of oligonucleotides to develop an RNA-based Oligo-PROTAC and a brain-penetrant DNA nanoflower functionalized with hundreds-to-thousands of transferrin receptor (TfR)-targeting aptamers. The DNA nanoflower also incorporates multiple oligonucleotide binding sites, enabling efficient loading of the Oligo-PROTAC. This nanoplatform, termed FRONTAC, facilitates transport across the blood–brain barrier (BBB) and promotes the selective degradation of disease-associated proteins. As a proof of concept, they designed a FRONTAC targeting FUS (fused in sarcoma, abnormal protein aggregates by RNA binding protein), demonstrating the potential of this technology as a versatile strategy for the targeted degradation of pathogenic proteins in the central nervous system [74]. Taken together, these findings provide new insights for the development of PROTACs, highlighting the importance of incorporating chemical features that facilitate receptor-mediated CNS uptake alongside traditional physicochemical optimization approaches.

## 5. Concluding Remarks and Perspective

Neurodegenerative diseases are defined by a progressive degeneration of neurons of both the central nervous system (CNS) and peripheral nervous system (PNS). Over the past few decades, our knowledge of protein aggregation and its role in these diseases has increased significantly. Among these diseases, Alzheimer’s disease (AD) represents a devastating neurodegenerative disorder. Because of the multifactorial nature of neurodegenerative disease, researchers envisaged the so-called multi-target approach, looking for drug candidates able to control multiple targets at the same time (multi-target-directed ligands, MTDLs), gaining an effective therapeutic action from their multiple activities. Proteolysis-Targeting Chimeras (PROTACs) are bivalent ligands that induce proximity between a protein of interest (POI), the target protein to be inhibited, and a ubiquitin E3 ligase. This recognition process initiates the polyubiquitination responsible for the proteasomal degradation of the target protein. The common structural scaffold of PROTACs consists of a POI binder linked to known ligands for E3 ligase with different spacers. The development of new multi-target-directed PROTACs aims for applications in the treatment of Alzheimer’s disease. This new medicinal chemistry approach will allow durable pharmacological effects compared to classical inhibitors, whose effects are limited to target occupancy. A multi-target-directed PROTAC could regulate the expression of neurodegenerative disease-associated proteins, achieving not only inhibition but also the complete degradation of target enzymes crucial for neurodegenerative disease onset and progression. The main goal will consist of the development of these innovative drugs, prioritizing pharmacokinetic and formulative studies with preclinical aims. In Table 2, we reported details regarding 15 selected PROTACs: the target, the E3 ligase, the evaluation of BBB permeation, the evaluation of degradation potency and the status of clinical trials that could be useful for the readers. At a glance, only a single ND PROTAC has progressed to clinical trials, underscoring the challenges in the pharmaceutical development of these molecules.

Indeed, bringing PROTACs from the laboratory to clinical trials involves navigating complex technical bottlenecks. A major hurdle stems from their chemical structure, where high molecular weight (>800 Da) and marked hydrophobicity restrict cell membrane permeability and lower overall bioavailability. Furthermore, high concentrations of PROTACs can trigger the “hook effect,” a phenomenon where non-productive binary complexes form instead of the active ternary complexes required for protein degradation. 

Beyond molecular design, PROTACs present distinct pharmacokinetic and safety challenges. Their large, bifunctional design complicates absorption, distribution, metabolism, and excretion, often leading to poor cellular uptake and rapid clearance. Unintended off-target degradation can also occur through non-specific binding, posing toxicity or immunogenicity risks that standard proteomics struggle to detect. Translating findings to humans is further complicated by species differences in E3 ligase expression between rodents and humans, while the potential degradation of metabolic enzymes creates unpredictable drug–drug interactions when co-administered with other therapies. 

Overcoming these preclinical hurdles requires a combination of advanced methods and strategic planning. Integrating comprehensive in vitro and in vivo PK/PD modeling helps researchers accurately predict drug behavior and optimize dosing schedules. To verify therapeutic efficacy and specificity, robust target engagement assays such as mass spectrometry and Western blotting are essential. Developers can also improve translatability by employing humanized mouse models that express human E3 ligases alongside human cell lines, while early DDI screening against key enzymes like cytochrome P450 mitigates clinical safety risks [75]. Finally, partnering with specialized preclinical testing laboratories provides the necessary technical expertise and regulatory guidance to streamline the entire process. Combining these modern delivery technologies, rigorous preclinical evaluations, and expert collaborations will ultimately unlock PROTACs’ full potential to treat complex diseases.

## Figures and Tables

**Table 1:** Selected ND PROTAC activity and cell permeation results.

			Permeability			
CMPD	Target	Effect	In Vitro	In Vivo	Results	Ref.
**1** (HyT-Tau-CPP)	Tau	Reduces Tau levels; in vitro and in vivo assays.	Flow cytometry (N2a cells)	---	High ability to get into cells	[42]
**2** (C004019)	Tau	Reduces Tau levels; in vitro and in vivo assays.	---	Time-dependent concentration after subcutaneous administration (wild-type mouse)	Maximal brain concentration (10.8 ng/mL)	[44]
**3** (I3)	Tau	Degrades Tau protein; in vitro assay	---	Brain-to-plasma concentration ratio ≥ 1.60 in healthy rats; administration (po)	Brain-penetrant compound.	[45]
**4** (PT-65)	GSK3	Potent degradation against GSK3α and β; in vitro and in vivo models	PAMPA (Pe = 0.27 × 10^−6^ cm/s)	---	---	[48]
**5**	GSK3	Decreases GSK3-β level in vitro	PAMPA-BBB (Pe = 15.33 × 10^−6^ cm/s)	---	CNS ± permeable	[49]
**6** (KH1)	GSK3	Almost-complete GSK3α and GSK3β degradation in vitro	---	Brain-to-plasma concentration ratio = 0.18 (iv in female Balb/c mice)	Moderate brain penetration (concentration 16 nM at 2 h)	[50]
**7**	α-Syn	Degradation rate of 89% in vitro; dose-dependent degradation in vivo	Confocal LSM (HEK293T cells)	---	Good membrane-penetrating ability	[54]
**8** (XL01126)	LRRK2	In vitro extensive LRRK2 degradation in different cell lines	Caco-2 permeability (A–B < 0.74 × 10^–6^ cm/s B–A < 1.43 × 10^–6^ cm/s)	Brain-to-plasma concentration ratio <0.035 (po; iv; ip, mice)	Both orally bioavailable and BBB-permeable (but low concentration in the brain and in CSF)	[57]
**9**	mHtt	Significant mHtt and wtHtt degradation in vitro	---	---	---	[61]
**10**	mHtt	Significant mHtt and wtHtt degradation in vitro	---	---	---	[61]
**11**	mHtt	Successful mHtt and wtHtt degradation in vitro; less efficacy than compound 9 or 10.	---	---	---	[62]
**12** (D4)	TDP-43	TDP-43 degradation; in vitro and in vivo models	Flow cytometry (N2a cells)	---	Penetration into cells in a short time	[64]
**13** (JMF 4560)	TDP-43	Significant C-TPD without affecting endogenous full-length TDP-43; in vitro and in vivo models	---	---	---	[65]
**16** (T3)	α-Syn and Tau	Significant and simultaneous α-Syn and Tau degradation in vitro	BBB-model (Confocal LSM, SH-SY5Y cells)	Brain fluorescence imaging (iv, mice)	Penetration correlated positively with the administered dose	[66]

**Table 2:** Summary table comparing selected ND PROTACs.

Compound	Target	E3 Ligase	Cell or BBB Penetration	Degradation Potency (DC_50_/D_max_), [IC_50_, POI] ^a^	Clinical Status
**1** [42] (HyT-Tau-CPP)	Tau	Hydrophobic tag motif (HyT)	N2a cell-permeant	80% in a dose-dependent manner (Tau-EGFP-overexpressing cells)	n.d.
**2** [44] (C004019)	Tau	VHL (AHPL)	maximal concentration at 10.8 ng/mL at 0.167 h with t _1/2_ of 1.29 at 3 mg/kg of subcutaneous administration and a brain/plasma ratio at the maximal concentration of 0.00866	[IC_50_ = 0.00785 μM] (HEK293-hTau cells)	n. d.
**3** [45] (I3)	Tau	CRBL (Thalidomide)	favorable brain penetration; brain-to-plasma concentration ratio exceeding 1.6 in healthy rats at 30 mg/kg (po) administration	n.d.	n.d.
**4** [48] (PT-65)	GSK-3	CRBN (Pomalidomide)	PAMPA: Pe = 0.27 × 10^−6^ cm/s, calculated value: LogP_o/w_ = 1.61, and Bioavailability score = 0.17 (Swiss ADME)	GSK3α: DC_50_ = 28.3 nM, GSK3β: DC_50_ = 34.2 nM (SH-SY5Y cell lines)	n.d.
**5** [49]	GSK-3	CRBN (Pomalidomide)	moderate BBB permeability, Pe = 15.33 ± 1.12 × 10^−6^ cm/s (PAMPA-BBB)	GSK-3β: DC_50_ of 6.22 μM (SH-SY5Y cell line)	n.d.
**6** [50] (KH1)	GSK3	CRBN	oral bioavailability of 1.6%, plasma concentration above 88 nM, brain concentration of 16 nM and a brain/plasma ratio of 0.18 (at 2 h following i.v. dosing at 0.37 mg/kg, Balb/c mouse)	DC_50_ values in the picomolar to single-digit nanomolar range for degrading both GSK-3.	n.d.
**7** [54]	α-syn	CRBN (Pomalidomide)	confocal LSM with 10 μM on HEK293T cells (live cells); good membrane-penetrating ability	DC_50_ = 7.51 μM	n.d.
**8** [57]	LRRK2	VHL	high concentrations in plasma were achieved in all routes and detected in brain tissue and CSF at levels above the DC_50_	DC_50_ values in multiple cell lines within 15–72 nM, D_max_ values from 82 to 90% and degradation half-life from 0.6 to 2.4 h	n.d.
**AVR-102** [34,58]	LRRK2		crosses the blood–brain barrier	[IC_50_ = 0.14 nM]	Phase I
**9** [61]	mHtt	cIAP1 (BE04)	n. d.	n. d.	n. d.
**10** [61]	mHtt	cIAP1 (BE04)	n. d.	n. d.	n. d.
**11** [62]	mHtt	cIAP1 (MV1)	n. d.	n. d.	n. d.
**12** [64] (D4)	TDP-43	Hydrophobic tag motif (HyT)	flow cytometric results: could penetrate into cells	n.d.	n.d.
**13** [65] (JMF 4560)	TDP-43	CRBN (Pomalidomide)	n.d.	Reduction in aggregate levels (0.41 ± 0.06) compared with control sample (1.08 ± 0.31)	n.d.
**16** [66] (T3)	α-syn Tau	Thalidomide	penetration correlated positively with the administered dose	α-syn: DC_50_ = 1.57 μM, D_max_ = 78%, Tau: DC_50_ = 4.09 μM, D_max_ = 61%	n.d.

^a^ When the degradation potency was not published, the IC_50_ of the POI was reported.

## Data Availability

No new data were created or analyzed in this study. Data sharing is not applicable to this article.

## Abbreviations

The following abbreviations are used in this manuscript:

PROTAC	Proteolysis-Targeting Chimera
TPD	Targeted protein degradation
AD	Alzheimer’s disease
PD	Parkinson’s disease
POI	Protein of interest
VHL	von Hippel–Lindau
CRBN	Cereblon
bRo5	Beyond Rule-of-Five
GSK-3	Glycogen synthase kinase 3
